# HLA Class I-T Cell Epitopes from *trans*-Sialidase Proteins Reveal Functionally Distinct Subsets of CD8^+^ T Cells in Chronic Chagas Disease

**DOI:** 10.1371/journal.pntd.0000288

**Published:** 2008-09-03

**Authors:** María G. Alvarez, Miriam Postan, D. Brent Weatherly, María C. Albareda, John Sidney, Alessandro Sette, Carina Olivera, Alejandro H. Armenti, Rick L. Tarleton, Susana A. Laucella

**Affiliations:** 1 Hospital Interzonal General de Agudos “Eva Perón”, San Martín, Provincia de Buenos Aires, Argentina; 2 Instituto Nacional de Parasitología “Dr. Mario Fatala Chabén”, Buenos Aires, Argentina; 3 Center for Tropical and Emerging Global Diseases, University of Georgia, Athens, Georgia, United States of America; 4 La Jolla Institute of Allergy and Immunology, La Jolla, California, United States of America; Hospital Universitário, Brazil

## Abstract

**Background:**

Previously, we identified a set of HLA-A020.1-restricted *trans*-sialidase peptides as targets of CD8^+^ T cell responses in HLA-A0201^+^ individuals chronically infected by *T. cruzi*.

**Methods and Findings:**

Herein, we report the identification of peptides encoded by the same *trans*-sialidase gene family that bind alleles representative of the 6 most common class I HLA-supertypes. Based on a combination of bioinformatic predictions and HLA-supertype considerations, a total of 1001 epitopes predicted to bind to HLA A01, A02, A03, A24, B7 and B44 supertypes was selected. Ninety-six supertype-binder epitopes encoded by multiple *trans*-sialidase genes were tested for the ability to stimulate a recall CD8^+^ T cell response in the peripheral blood from subjects with chronic *T. cruzi* infection regardless the HLA haplotype. An overall hierarchy of antigenicity was apparent, with the A02 supertype peptides being the most frequently recognized in the Chagas disease population followed by the A03 and the A24 supertype epitopes. CD8^+^ T cell responses to promiscuous epitopes revealed that the CD8^+^ T cell compartment specific for *T. cruzi* displays a functional profile with T cells secreting interferon-γ alone as the predominant pattern and very low prevalence of single IL-2-secreting or dual IFN-γ/IL-2 secreting T cells denoting a lack of polyfunctional cytokine responses in chronic *T. cruzi* infection.

**Conclusions:**

This study identifies a set of *T. cruzi* peptides that should prove useful for monitoring immune competence and changes in infection and disease status in individuals with chronic Chagas disease.

## Author Summary

At present, 16–20 million people in Central and South America are infected with *Trypanosoma cruzi*, the causative agent of Chagas disease in humans. The primary clinical consequence of the infection is a cardiomyopathy, which manifests in approximately 30% of infected individuals, many years after the initial infection. Our work in Chagas disease patients began as an effort to assess the range and specificity of antigens that were recognized by T cells, in particular CD8^+^ T cells, in individuals with long-term infections with *Trypanosoma cruzi*. Trans-sialidase proteins from *T. cruzi* are major surface and released proteins that are targets of humoral and cellular immune responses. We previously, identified a set of *trans*-sialidase peptides that were recognized by a very low frequency of chronically *T. cruzi*-infected subjects. Based on bioinformatic predictions, herein we report the identification of new *trans*-sialidase epitopes that are recognized by a higher proportion of *T. cruzi*-infected people. The functional profile of T cells specific for these peptides is characteristic of an infection with long term stimulation of the immune system, with high levels of IFN-γ-secreting T cells and low levels of IL-2 production. This set of *T. cruzi* peptides should prove useful for monitoring immune competence and changes in infection and disease status in individuals with chronic Chagas disease.

## Introduction

*Trypanosoma cruzi*, the causative agent of Chagas disease, constitutes a prominent human health problem in Central and South America [1]. It is estimated that approximately 4 million individuals have chagasic heart disease, making Chagas disease the most frequent cause of infectious cardiomyopathy in the world [2].

Immune control of *T. cruzi* is complex, requiring the generation of a substantial antibody response and the activation of both CD4^+^ and CD8^+^ T cell responses. The intracellular replication and localization of *T. cruzi* have focused attention on CD8^+^ T cell responses as a critical component of protective immunity in *T. cruzi* infection [3],[4]. Even in cases in which such responses are stimulated sufficiently to control the acute infection, *T. cruzi* is not completely cleared but instead, persists in infected hosts for decades. The inflammatory responses that such persistence induces may eventually result in the tissue damage that is associated with chronic Chagas disease [5],[6]. Supporting the hypothesis that disease is less severe when immune control is more efficient we have shown that chronic chagasic subjects with no or mild clinical disease have significantly higher frequency of interferon-gamma (IFN-γ) producing T cells specific for *T. cruzi* than do individuals with more severe disease [7],[8]. This apparent impairment in CD8^+^ T cell responses was associated with an increased frequency of fully differentiated memory (CD27^−^CD28^−^CD45RA^−^) CD8^+^ T cells and a high rate of apoptosis, possibly reflecting exhaustion in the CD8^+^ T cell compartment [8]. The loss of parasite-specific T cell responses during chronic infection, together with the diversity of HLA types and the diversity of parasite strains and thus epitope expression, have made it difficult to identify the specific epitopes recognized by *T. cruzi*-specific CD8^+^ T cells, and therefore to quantify the response and function of parasite-specific CD8^+^ T cells from chronic chagasic individuals.

A number of parameters have been implicated in determining which pathogen epitopes are primarily targeted by T cell responses, including the nature of the restricting MHC allele, efficiency of epitope processing and translocation into the endoplasmatic reticulum, the degree of sequence variability in epitopes derived from highly variable pathogens and antigen availability by either cross-presentation of exogenous antigen or processing of intracellular proteins [9]–[11]. In addition, interactions among different T cell populations and cross-reactivity between “self” and/or “other” pathogen antigens, as well as the presence of antagonistic epitopes, may further impact immunodominance patterns [12].

*Trans*-sialidases (ts) are a large family of surface molecules (1430 genes annotated [13]) previously shown to be the targets of antibody responses [14]–[17], CD4^+^ T cells [18]–[20] as well as CD8^+^ T cells in mice [19]–[22] and in humans with chronic *T. cruzi* infection [7], [23]–[25]. We have recently reported on a large-scale screen for dominant parasite peptides recognized by CD8^+^ T cells in *T. cruzi* infected mice, showing that the CD8^+^ T cell response is highly focused on epitopes encoded by members of the large ts family of genes [25]. Contrasting with the mouse system, a lower and more diffuse pattern of CD8^+^ T cell responses specific for HLA-A0201-restricted ts peptides in human subjects was found [25]. An additional complication of translating the results of studies in mice to the assay of human T cell responses is the large degree of polymorphism of human HLA alleles and the requirement to HLA-type each individual subject prior to attempting to measure T cell responses.

Class I molecules can be grouped based upon structural similarities, into sets (supertypes) with similar predicted binding motifs; nine such supertypes have been defined to date [26]. The six most common HLA supertypes cover one or more HLA allele in >95 of a human population, irrespective of ethnicity [26],[27]. Thus, by defining a set of pathogen peptides that are presented by these six HLA supertypes, one can screen T cell responses in a majority of individuals without having to conduct HLA typing and tailoring the peptide set to the individual HLA type [28],[29].

The present study was conducted to extend the identification of immunodominant CD8^+^ T cell epitopes in *T. cruzi* by searching promiscuous HLA-Class I epitopes from ts proteins based on HLA-supertype binding predictions and the frequency of representation of the peptides in the predicted *T. cruzi* proteome, and to investigate the functional phenotype of T cells from chronic Chagas disease patients in response to the selected peptides.

This analysis provided the precise identity and promiscuity of a set of new ts epitopes based upon binding to the 6 most common class I HLA-supertypes. The simultaneous assessment of IFN-γ and IL-2 production after stimulation with ts-derived peptides, demonstrates that the CD8^+^ T cell compartment specific for *T. cruzi* displays a very low level of polyfunctional cytokine responses with a predominance of T cells secreting IFN-γ alone and very low prevalence of single IL-2-secreting or dual IFN-γ/IL-2 secreting T cells.

## Materials and Methods

### Selection of study population

*T. cruzi*-infected adults volunteers aged 29 to 61 were recruited at the Chagas disease Section of the Cardiology Department, Hospital Interzonal General de Agudos “Eva Perón”, Buenos Aires, Argentina. These individuals were originally infected while living in areas endemic for *T. cruzi* but had lived in this non-endemic area for a minimum of 15 years. Signed informed consent was obtained from all individuals prior to inclusion in the study. *T. cruzi* infection was determined by indirect immunofluorescence assay, hemagglutination, and ELISA techniques [30]. Chronic chagasic subjects were evaluated clinically and grouped according to the Kuschnir grading system [31]. Group 0 (G0, n = 37; mean age±SD = 46±8 y) included seropositive individuals exhibiting a normal electrocardiography (ECG), and a normal chest-X ray; group 1 (G1, n = 8; mean age±SD = 50±2 y) seropositive patients had a normal chest-x ray but abnormalities in the ECG; group 2 (G2, n = 6; mean age±SD = 48±9 y) seropositive patients had ECG abnormalities and heart enlargement as determined by chest X-ray and group 3 (G3, n = 4) seropositive patients had ECG abnormalities, heart enlargement and clinical or radiologic evidence of heart failure. Patients had not received treatment for *T. cruzi* at the time of sample collection.

The uninfected control group (n = 10) consisted of age-matched Caucasian of areas where *T. cruzi* was not endemic who were found to be negative for *T. cruzi* by serologic testing. *T. cruzi*-infected subjects and uninfected controls with hypertension, ischemic heart disease, cancer, HIV infection, syphilis, diabetes, arthritis or serious allergies were excluded from this study. This study was approved by the Institutional Review Boards of the Hospital Interzonal General de Agudos “Eva Perón” Buenos Aires, Argentina and the University of Georgia, Athens, GA, USA.

### Collection of peripheral blood mononuclear cells (PBMCs)

Approximately 50 ml of blood were drawn by venipuncture into heparinized tubes (Vacutainer, Becton-Dickinson, San José, CA). PBMC were isolated by density gradient centrifugation on Lymphocyte Separation Medium (ICN, Ohio,OH) and resuspended in RPMI (Mediatech, Herndon, VA) supplemented with 10% heat-inactivated FCS (Hyclone Laboratories, Logan, UT).

### Peptides

HLA supertype binding affinity predictions were performed computationally [27] on ts proteins using the raw *T. cruzi* genome sequencing reads (*T. cruzi* Genome Sequencing Consortium). Nine to 10 aa-length peptides were synthesized by Pepscan Systems (Lelystad, the Netherlands). Lyophilized peptides were dissolved at 10–20 mg/ml in DMSO, aliquoted and stored at −20°C.

### *T. cruzi* lysate

Protein lysate from *T. cruzi* amastigotes was obtained by 4 freeze/thaw cycles followed by sonication as previously reported [7]. Briefly, trypomastigotes from Brazil strain were cultured overnight in pH 5 DMEM (Mediatech) to transform trypomastigotes to amastigotes. After washing, the parasites were frozen at −20°C and thawed twice. Thereafter, the sample was subjected to 2 freeze/thaw cycles at −70°C followed by sonication. The supernatant of a 12000 rpm centrifugation was collected, filter-sterilized and the protein concentration determined.

### HLA-A0201 typing

HLA-A0201 typing was performed as previously described [7] by incubating 1×10^6^ PBMC with HLA-A2.1-specific mAB BB7.2 [32] (10 µg/ml; American Type Culture Collection, Bethesda, MD) for 30 min at 4°C, followed by FITC-labelled F(ab')_2_ goat anti-mouse IgG (1/50 dilution; Immunotech, Marseille, France). Positive fluorescence indicating expression of HLA-A2.1 was assessed by analysis of cells using fluorescence microscopy.

### HLA-A class I binding assays

The binding affinity of synthetic peptides for HLA class I molecules was quantified by measuring their ability to competitively inhibit the binding of a radiolabeled standard probe peptide to purified detergent-solubilized HLA-A2.1 molecule, as previously described [27],[32].

### IFN-*γ* and IL-2 ELISPOT assays

The number of peptide-specific IFN-γ and IL-2-secreting T cells was determined by *ex vivo* ELISPOT using commercial kits (Becton & Dickinson, San Diego, CA). Cryopreserved PBMC from chronic chagasic subjects were seeded in triplicate wells at a concentration of 4×10^5^/well, and stimulated with individual peptides (10 µg/ml), peptide pools (7–13 peptides per pool, 5 µg/ml/peptide) comprising HLA A01 (2 pools), A02 (2 pools), A03 (2 pools), A24 (2 pools), B07 (1 pool) or B44 (1 pool)-binding peptides or *T. cruzi* lysate (10 µg/ml) for 16–20 hr at 37°C and 5% CO_2_. For controls, cells were incubated with complete RPMI, A2-restricted HIV Pol (amino acids 510–518: ILKEPVHGV), A2-restricted flu matrix 1 (amino acids 58–66: GILGFVFTL) or 50 ng/ml pokeweed mitogen and 500 nM ionomicin (Sigma). Spot forming cells (SFCs) were automatically enumerated using ImmunoSpot analyzer (CTL).

The mean number of SFCs in triplicate wells was obtained for each condition, and the number of specific IFN-γ and IL-2-secreting T cells (net SFCs), was calculated by subtracting the value of the wells containing media alone from the peptide/mitogen-stimulated spot count. Responses were considered significant if a minimum of 25 SFCs/1×10^6^ PBMC were present per well, and additionally, this number was at least twice the value of wells with media alone [33].

### Intracellular IFN-*γ* and IL-2 staining

IFN-γ and IL-2 production was determined by intracellular staining after PBMCs isolated from subjects with chronic *T. cruzi* infection were stimulated with 15 µg/ml of *T. cruzi* lysate, 5 µg/ml of A02-restricted peptide pool or media alone, for 16–20 h. A final concentration of 10 µg/ml brefeldin A was added for the last 10 h of incubation. One million PBMCs were stained with anti-CD8 (APC) and anti-CD4 (PerCP) for 30 min at 4°C. The cells were then fixed and permeabilized with Cytofix/Cytoperm solution (Becton & Dickinson) for 15 min at 4°C, followed by 2 washes with Perm/Wash solution (Becton & Dickinson). After washing, the cells were stained with a FITC-conjugated anti-human IFN-γ (FITC) and IL-2 (PE) for 30 min at 4°C and were analyzed by use of a FACScalibur (Becton Dickinson Immunocytometry Systems).

Acquisition and analysis of data were performed with CellQuest software. Typically, 500,000 lymphocytes were collected by gating on forward-light scatter versus side-light scatter. Data were reported as percentage of the gated population expressing IFN-γ and IL-2. T-cell responses directed against antigens were considered positive if the frequency of cytokine-producing T cells was threefold higher than the frequency with medium alone.

### Statistical analysis

Comparisons of the frequencies of responders among the different clinical groups were evaluated by use of the χ^2^ test and Fisher's exact test. Differences were considered to be statistically significant at P<0.05.

## Results

### Identification of promiscuous HLA-A02-restricted epitopes derived from ts proteins

Based on the frequency of representation in the *T. cruzi* genome and HLA-A0201 binding efficiency, we have recently identified a set of epitope targets of memory CD8^+^ T cell responses in chronic chagasic subjects without manifest cardiac disease [25].

Taking into account that several studies have documented a correlation between immunogenicity and the capacity to bind multiple HLA molecules from a given supertype [28], [34]–[37], the affinity binding of these ts peptides to the other 4 most common HLA-A02 supertype alleles (A*0201, A*0203, A*0206 and A*6802) was measured to address whether these ts peptides displayed degenerate HLA-A2 binding capacity (Table 1). A range of binding activities was observed for the panel of peptides, from those that bound only one allele to those exhibiting high binding for all 5 alleles tested. Overall, a majority of the peptides were moderately degenerate, with 24 out of the 28 peptides binding 3 or more HLA-A02-supertype alleles (median±SD = 4,11+1,29 peptides bound).

**Table 1:** CD8^+^ T cell responses specific for ts-derived peptides with high representation in the *T. cruzi* genome in HLA-A2.1^+^ patients with chronic Chagas disease, measured by IFN-γ ELISPOT.

Peptide (A)	Sequence	N° genes (B)	N°proteins (C)	A2.1-binding		No. of positive responders/total no. of subjects assayed (%)		
				(IC 50 nM)	(D)	G0	G1	G2/G3
Ts 48	FANYNFTLV	253	51	0.47	(5)	3/10 (30)	0/7	1/14 (7)
Ts 3	GLLPSLLLLL	104	23	3.1	(4)	4/11 (36)	0/9	0/14
Ts 13	LLGLWGTAAL	63	14	499	(3)	2/9 (22)	0/6	1/15 (7)
Ts 2	LLGLWVFAAL	46	8	36	(4)	4/10 (40)	0/8	1/16 (6)
Ts 46	FANHNFTLV	39	6	119	(5)	3/11 (27)	1/8 (13)	1/14 (7)
Ts 44	FANYKFTLV	36	9	49	(5)	7/15 (47)	2/12 (17)	1/17 (6)
Ts 43	FANNKFTLV	36	17	116	(5)	2/9 (22)	0/6	1/11 (9)
Ts 58	RVSRPTTVV	32	5	2487	(2)	0/6	0/5	0/8
Ts 66	DVSRPTAVV	21	7	>50000	(1)	3/7 (43)	1/6 (17)	0/9
Ts 37	FVNHRFTLV	18	6	304	(5)	6/11 (44)	2/9 (22)	2/17 (12)
Ts 51	FANYNFTLL	17	2	1499	(2)	0/7	0/5	0/10
Ts 52	ELLRPTTLV	16	6	2407	(1)	1/7 (14)	0/5	0/10
Ts 49	FANNEFTLV	13	5	47	(5)	5/11 (45)	1/9 (11)	2/15 (13)
Ts 68	FTNNKFTLS	12	0	>50000	(3)	0/6	0/5	0/8
Ts 38	FANHKFTLV	10	4	148	(4)	5/16 (31)	2/12 (17)	2/17 (12)
Ts 35	FVSCDFTIV	10	0	30	(5)	6/16 (38)	3/11 (27)	2/16 (13)
Ts 24	FLSHDFTLV	7	4	0.46	(4)	4/10 (40)	0/8	1/15 (7)
Ts 6	LLGLWGLATA	7	4	24	(5)	0/9	0/6	2/13 (15)
Ts 4	RLLPSLLLLL	7	2	0.80	(4)	1/10 (10)	0/6	1/14 (7)
Ts 28	FVNYDFALV	6	0	79	(5)	4/11 (36)	0/8	2/15 (13)
Ts 32	FANCNFTLV	5	1	79	(5)	5/15 (33)	0/11	2/16 (12)
Ts 50	FVNYDFTIV	3	0	77	(5)	2/9 (22)	0/6	1/13 (8)
Ts 47	FVDYNFSLV	3	0	1.1	(5)	2/9 (22)	0/6	1/12 (8)
Ts 45	FVNYNFTLV	2	1	0.25	(5)	3/13 (23)	1/9 (11)	1/15 (7)
Ts 56	IANYNFTLV	1	0	29640	(3)	1/6 (17)	1/4 (25)	0/9
Ts 27	FLSHNFTLVF	0	0	0.61	(5)	2/9 (22)	1/6 (17)	1/14 (7)
Ts 26	SLSHYFTLVF	0	0	1.6	(5)	5/15 (33)	1/11 (9)	1/17 (6)
Ts 34	FANHDFTLV	0	0	101	(5)	2/9 (22)	1/6 (17)	1/13 (8)
		Total no. of responders				9/15*(60)	5/12 (42)	4/18 (22)
		Mean net SFCs±SD /10^6^ PBMC in peptide-stimulated wells(E)				120±185	120±105	100±45
		Mean SFCs±SD/10^6^ PBMC in media alone				18±16	23±28	18±26

(A) All the peptides assayed were recognized by spleen cells from *T. cruzi*-infected A2 transgenic mice (25).

(B) Number of annotated *T. cruzi* genes containing indicated epitopes (13).

(C) Number of proteins containing indicated epitopes identified in *T. cruzi* proteome (59).

(D) Number of alleles of the HLA-A2 supertype bound.

(E) Mean values was obtained from responders subjects. (^*^) P = 0.04 vs. G2/G3 (Fisher's exact test).

### IFN-γ ELISPOT responses to MHC I supertype-restricted epitopes derived from ts proteins

To extend coverage of HLA types in the Chagas disease population, we next attempted to identify peptides with high binding affinity for additional HLA class I supertypes. Because our previous work in a murine model of Chagas disease had shown that CD8^+^ T cell responses were focused heavily on peptides encoded by multiple ts family genes [25], we restricted our analysis in humans to ts peptides as well.

HLA supertype binding affinity predictions were performed on ts sequences using the raw *T. cruzi* genome sequencing reads, yielding over 20,000 putative epitopes with a predicted IC_50_ (50% inhibitory concentration) of less than 100 nM. Only those peptides with at least 100 occurrences in the ∼1.3 million sequence reads were further analyzed yielding a total of 1001 epitopes predicted to bind to HLA A01, A02, A03, A24, B07 or B44 supertypes (Table 2). The supertype for which the fewest peptides were predicted was B7, reflecting the fact that this supertype is associated with a stringent requirement for the relatively infrequent proline residue in position 2 of its peptide ligands [26]. At the other end of the spectrum, A2 and A3 supertypes have the largest number of peptides, reflecting the relative commonality of the residues associated with these motifs (Table 2).

**Table 2:** *T. cruzi*-specific IFN-γ ELISPOT responses to ts-derived peptides restricted by MHC class I supertypes.

Class I supertype (*)	Predicted no. of peptides	No. of positive responders/total no. of subjects evaluated (%)	Net SFCs per 10^6^ PBMC (#)
HLA A01	116	2/25 (8)	25–50
HLA A02	451	7/25 (28)	15–500
HLA A03	287	6/25 (24)	25–53
HLA A24	100	4/25 (16)	18–40
HLA B07	20	2/25 (8)	15–18
HLA B44	27	2/25 (8)	20–25
**Total**	**1001**	**13/25 (52)**	

(^*^) Peripheral blood mononuclear cells were stimulated with pooled peptides from each HLA-type; SFC: spot forming colony; (#) The number of spots from wells containing media alone was subtracted from the peptide-stimulated spot count, mean SFCs±SD/10^6^ PBMC in media alone = 9±12.

**Figure 1:**
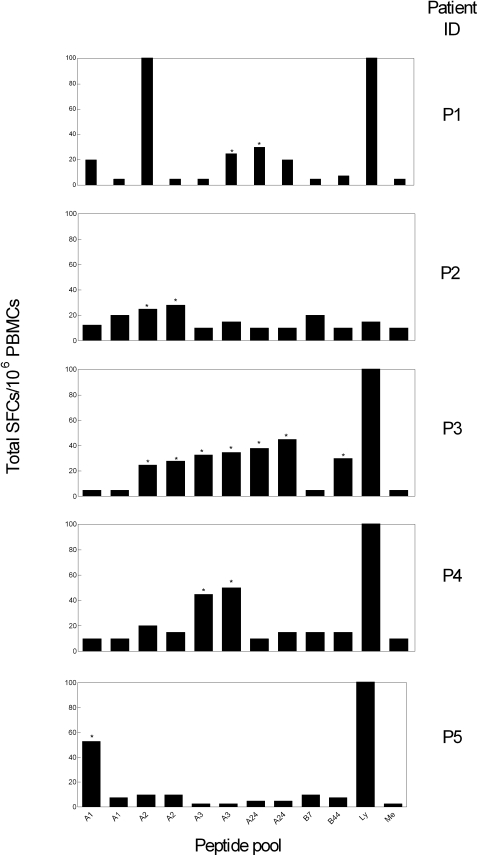
IFN-γ ELISPOT responses to ts proteins of *T. cruzi* restricted by class I HLA supertypes. PBMC from chronic Chagas disease subjects were stimulated in bulk with HLA-A01, HLA-A02, HLA-A03, HLA-A24, HLA-B07 and HLA-B44 supertype ts peptide pools, *T. cruzi* lysate (Ly) or media (Me) alone and tested for IFN-γ secretion as described in the Materials and Methods. Representative positive responses for single subjects are shown. (*) Indicates positive responses as defined in the Materials and Methods.

The number of annotated *T. cruzi* genes containing supertype-binding epitopes was then used to prioritize peptides to be assessed for recognition by CD8^+^ T cells from a group of non-HLA-typed chagasic subjects (Table 2). IFN-γ ELISPOT responses to peptide pools containing a total of ninety-six epitopes representing HLA A01 (18 epitopes), HLA A02 (20 epitopes), HLA A03 (20 epitopes), HLA A24 (14 epitopes), HLA B07 (11 epitopes) and HLA-B44 (13 epitopes) supertypes were evaluated in 25 chronic Chagas disease subjects without clinical and radiological evidence of heart disease (G0 status) regardless of their HLA type. Thirteen out of 25 (52%) subjects showed positive responses to supertype-restricted ts-sialidase peptides ranging in magnitude from 15 to 500 *T. cruzi*-specific SFCs/10^6^ (SFCs in media alone subtracted, Table 2). Four subjects had positive responses to more than one supertype (3 of these subjects recognized both HLA-A02 and HLA-A03-binding peptides, Fig. 1). It is noteworthy that responses to HLA-A02 and HLA03 supertype-restricted epitopes covered 77% of the total responses detected, consistent with the high worldwide frequency of these alleles [26].

As a positive control for the ability of T cells to respond to parasite antigens, the response to an amastigote lysate preparation which has been previously shown to activate both CD4^+^ and CD8^+^ T cell subsets in *T. cruzi*-infected mice [22] and subjects [7] was also measured. Twenty out of the 25 (80%) patients evaluated displayed positive responses to the lysate with 11 subjects responding to both the *T. cruzi* lysate and ts-derived peptides. Non-responders to ts peptides had either negative or very low responses to the amastigote lysate denoting the overall low *T. cruzi*-specific T cell responses in these individuals.

Next, selected positive peptide pools were deconvoluted to identify the individual peptides responsible for the activity detected within the pool. Stimulation of PBMC from 12 of the responder subjects with single peptides revealed a total of 27 distinct epitopes (1 HLA-A01, 16 HLA-A02 and 10 HLA-A03-binding peptides) as target epitopes (Table 3). Because of limitations of sample availability and the low number of subjects responsive to the A24, B07 and B44-binding peptide pools, the individual target epitopes for these supertypes were not determined.

**Table 3:** Summary of identified A1, A2 and A3 supertype epitopes.

Peptide sequence	HLA allele	No. Net IFN-γ SFCs/10^6^ PBMC (*)
VTDNNRSFY	A1	30
LWLTDNTHI	A2	463
LLLGLWGFA	A2	30
YNFTLVATV	A2	22
FTSAVLLLL	A2	25
MLVTLPVYS	A2	15
NVMLVTLPV	A2	18
FVSPSLVSA	A2	18
ALSSSLGNV	A2	22
HLFYSAVLL	A2	40
FLYNRPLNS	A2	18
HNFTLVASV	A2	15
LLLLVVMMCC	A2	18
TSAVLLLLVV	A2	18
SIPTAGLVAV	A2	22
FQGAWAEWPV	A2	32
RVLLLLLLGL	A2	20
MLSRVAAVK	A3	38
FTLVATVSI	A3	70
FTLVASVTI	A3	35
AVAEAQCKK	A3	18
VALMLQGNK	A3	18
LVTLPVYSK	A3	15
DVAASSLLY	A3	13
ITATIEGRK	A3	50
IYMLVGKYS	A3	65
GVIAAFAEGH	A3	22

(*): Representative results from single individuals are shown.

Net responses were calculated by subtracting the value of the wells containing media alone from the peptide-stimulated spot count; mean SFCs±SD/10^6^ PBMC in media alone = 13±10.

The nature of a T cell response to complexes formed by peptides with major histocompatibility proteins can be greatly affected by variations in the peptide's amino acid sequence [38],[39]. Some peptides (agonists) elicit the full range of known responses; others (partial agonists), often differing from agonists by only one or a few amino acid residues, elicit partial responses; while others (antagonist peptides) inhibit the activity of agonists. To rule out the possibility that pooling peptides might influence ELISPOT responses, PBMC from 9 non-responder subjects to pooled peptides were assayed with individual peptides. We found positive responses in only one subject with previous negative responses to pooled peptides demonstrating that pooling peptides had a minimal effect on detection of T cell responses.

Altogether, a hierarchy of antigenicity was apparent with the A02 supertype peptides being the most frequently recognized in the Chagas disease population followed by the A03 and the A24 supertype peptides. This analysis also allowed the identification of previously unknown ts epitopes that may be useful for the monitoring CD8^+^ T cell responses in Chagas disease subjects regardless of their HLA type.

### IFN-γ and IL-2 ELISPOT responses to ts-derived HLA-A201-binding peptides in HLA-A201^+^ *T. cruzi* chronically infected subjects with different degrees of cardiac dysfunction

The degenerate A02 peptides identified above were used to examine correlations between disease severity and CD8^+^ T cells responses in chronically *T. cruzi*-infected subjects. PBMCs from HLA-A201-positive *T. cruzi*-infected and uninfected individuals were tested for IFN-γ secretion in response to 28 individual HLA-A0201-binding peptides, showing 60% of individuals (9/15) in G0 responding and 22% of the patients (4/18) in G2–G3 responding to ts peptides (Table 1). The uninfected control group did not show positive responses to HLA-A02-binding peptides assessed in pools or separately (mean net SFCs±SD /10^6^ PBMC in peptide-stimulated wells = 1.52±2.01; mean SFCs±SD /10^6^ PBMC in media alone = 4.9 0±4.03). These results confirm our previous findings demonstrating that individuals with more severe disease had relatively poor T cell responses to whole parasite lysates [7].

**Figure 2:**
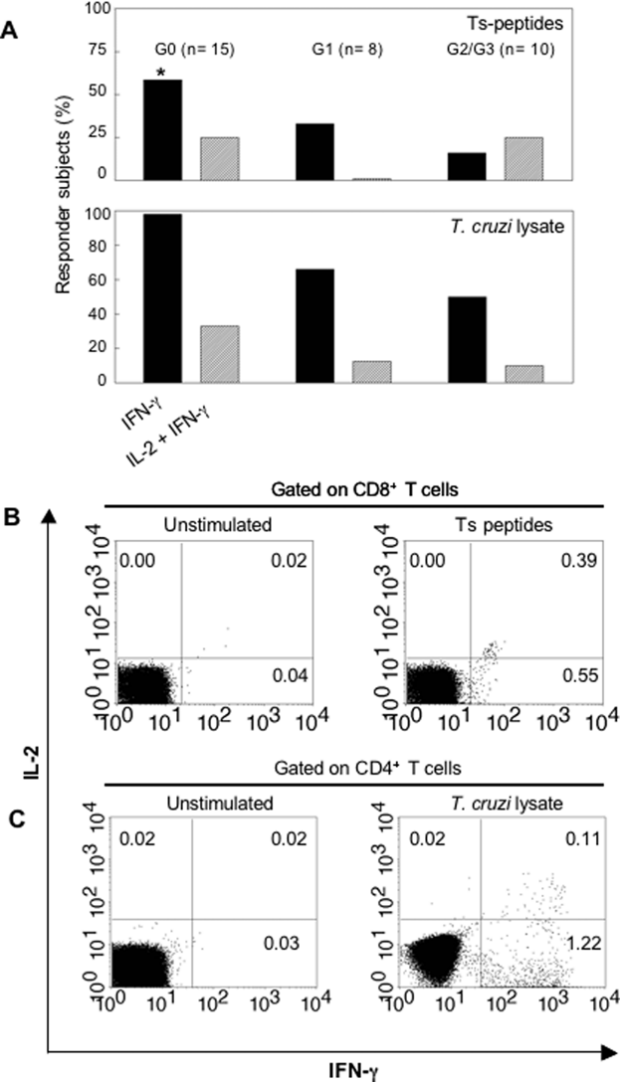
Analysis of functionally distinct populations of *T. cruzi*-specific T cells in HLA-0201^+^ chronic Chagas disease patients. PBMC from G0, G1 and G2/G3 patient groups were stimulated with 4 to 15 individual HLA-A0201-binding ts peptides (top) or with *T. cruzi* lysate (bottom) and tested for IFN-γ and IL-2 secretion by ELISPOT. The number of subjects with positive IFN-γ (black bars) or IFN-γ and IL-2 (hatched bars) ELISPOT responses out of the total evaluated ( percentage of responder subjects) is shown. (*) P = 0.035 vs G2/G3 (Fisher's Exact test) (A). Flow cytometry profiles of the distribution of IFN-γ and IL-2-secreting T cell populations. One representative profile is shown for CD8^+^ (B) and CD4^+^ (C) T cell responses after stimulation with ts peptides or *T. cruzi* lysate, respectively.

Although quantitative measures of T-cell function assessed by IFN-γ secretion have been proven to be a valuable tool for monitoring disease activity in various human infections, the measurement of other T-cell functions, such as IL-2 secretion, proliferation capacity and cytotoxic activity is also informative [40]. To further characterize the function of *T. cruzi*-specific T cells in subjects with chronic Chagas disease, IFN-γ and IL-2 secretion were simultaneously measured by ELISPOT after stimulation with 4 to 15 individual HLA-A0201- binding ts peptides or with a *T. cruzi* amastigote lysate preparation. Positive IL-2 responses were very low irrespective of the clinical status of the subjects, and was essentially undetectable in the G1 group upon stimulation with ts peptides (mean net SFCs±SD/10^6^ PBMC in subjects with positive IL-2 ELISPOT responses = 28±8, range = 15–55; mean SFCs±SD/10^6^ PBMC in media alone = 6±5), [Fig. 2A]. Less than 30% of the individuals evaluated displayed positive IL-2 and IFN-*γ* ELISPOT responses while IL-2 responses alone was not detected (Fig. 2A).

Low IL-2 production was not confined to the CD8^+^ T cell compartment as stimulation with a *T. cruzi* lysate, which had been previously shown to induce both CD4^+^ T and CD8^+^ T cell responses [7],^22^ also failed to induce IL-2 secretion (mean net SFCs±SD/10^6^ PBMC in subjects with positive IL-2 ELISPOT responses = 65±50, range = 25–153) [Fig. 2A]. In contrast, responses to influenza in subjects belonging to this same cohort showed a more functional response with 10 out of 15 responders to an A2-restricted Flu peptide producing both IFN-*γ* and IL-2 or IL-2 alone while the remaining 5 produced only IFN-*γ* ELISPOT responses (data not shown).

In order to determine if IFN-γ and IL-2- double-secreting T cells were present in these subjects, intracellular cytokine staining assays were performed with PBMC from 5 subjects with positive IL-2 and IFN-*γ* ELISPOT responses to *T. cruzi* antigens. In one individual, the recall T cell responses to the lysate were mainly composed of two functionally distinct populations, single IFN-*γ*-secreting and single IL-2-secreting T cells while in the 4 remaining individuals dual IFN-*γ*/IL-2-secreting cells and a larger set of single IFN-*γ*-secreting T cells was observed upon stimulation with a pool of HLA-A02-binding peptides (Fig. 2B) or a *T. cruzi* lysate (Fig. 2C).

This functional pattern of cytokine production in response to HLA-class I-restricted ts epitopes in conjunction with the negative correlation between disease severity and IFN-*γ*-secreting T cells, found in the present and former studies [7], as well the increased levels of fully differentiated T cells in these patients [8] supports the notion that the long-term persistence of *T. cruzi* infection may lead to a degradation of parasite-specific T cell responses.

## Discussion

Antigen-specific CD8^+^ T cells are crucial components of the immune response against a large number of pathogens, including *Trypanosoma cruzi* [21]. In human viral infections, reproducible measures of T cell function has led to the identification of functional signatures associated with disease control that are useful for the monitoring of disease activity [41]–[43] contrasting with the situation in human parasitic infections, mainly because of the difficulty in detecting the epitope specificity of CD8^+^ T cells in these chronic infections [7],[22],[24],[33],[44].

Previous studies of chronic chagasic subjects have shown that the frequency of *T. cruzi*-specific T cells recognizing HLA-A0201-restricted peptides from ts-sialidase, LYT-1 and calcium binding-proteins of *T. cruzi* was extremely low [7]. The prior analysis was restricted to HLA-A201-binding epitopes because of the high frequency of expression of this allele in individuals of various ethnic backgrounds [45],[46], including those living in areas of Latin America where Chagas disease is endemic [47], and thus the relative ease with which HLA-A201 expressing individuals can be identified. More recently, we applied a new strategy for the selection of potential epitopes target of CD8^+^ T cell responses in *T. cruzi* infection, based on both the high binding capacity for the HLA-A0201 allele and the high frequency of representation in the *T. cruzi* genome as the abundance of the source gene product appears to have a major contribution in immunodominance [48].

Even though HLA-A201 is expressed by ∼30% of individuals, this still leaves the majority of *T. cruzi*-infected subjects inaccessible to study T cell responses using defined CD8^+^ T cell epitopes. To extend the coverage of the patient population regardless of HLA type, herein we have focused on the identification of potentially immunogenic ts epitopes that bind to more than one MHC allele of the six most common HLA class I supertypes based on HLA-supertype binding predictions. The relatively high number of predicted epitopes for non-A02 supertypes was surprising considering that proteins “degenerate” for one supertype, are unlikely to also be degenerate for other HLA supertypes [28]. We show that promiscuous ts HLA-A02, HLA-A03 and HLA A24-binding epitopes are primary targets of memory CD8^+^ T cell responses in chronic Chagas disease patients, detecting responses in 13 out of 25 G0 subjects being the hierarchy in the antigenicity well correlated with the number of epitopes identified for these HLA supertypes. These results provide a specific set of class I MHC restricted peptides with which we can measure the frequency and functional activity of *T. cruzi*-specific CD8^+^ T cells in *T. cruzi*-infected individuals regardless of their HLA type.

In a previous study, we showed that 9 out of 10 (90%) asymptomatic (G0 clinical status) chronically infected subjects display positive CD8^+^ T cell responses to autologous *T. cruzi*-infected dendritic cells [8] while the use of an amastigote lysate preparation revealed CD8^+^ T cell responses in 6 out of 8 (75%) G0 subjects evaluated [7]. Even though we are testing only a fraction of all the potential class I-restricted CD8^+^ T cell epitopes herein, it is of significance that over one-half of the subjects evaluated displayed positive responses to peptides encoded by a single *T. cruzi* protein family, further supporting the immunodominant nature of ts gene products.

However, the pattern of recognition in subjects with chronic *T. cruzi* infections contrasts with the extremely strong and highly focused response to a few ts epitopes observed throughout *T. cruzi* infection in mice [25]. Similarly, anti-vaccinia responses in humans are distributed among several different determinants while a single epitope accounts for nearly one quarter of the total anti-vaccinia response in mice [49],[50]. Therefore, human responses against large complex pathogens are often multispecific and broad [10],[11]. A number of factors probably contribute to the greater variability of immunodominance hierarchies to ts epitopes in human infection with *T. cruzi*, including strain variance in ts genes, the influence of exposure to other infections [12],[51], heterozygocity in MHC genes and exhaustion of T-cell responses as a consequence of chronic antigen persistence [52]–[55].

Few reports have addressed the identification of targets of CD8^+^ T cells in the chronic phase of human *T. cruzi* infection [7],[24],[25],[56]. CD8^+^ T cell responses to HLA-A0201-restricted epitopes from the *T. cruzi* proteins cruzipain and FL-160 have been reported in a small group of chronically *T. cruzi*-infected individuals living in non endemic areas [24] and an HLA-A0201-binding peptide encoded by the kinetoplastid membrane 11 protein (KMP-11) has been shown to be the target of T cell responses in subjects living in areas endemic for *T. cruzi* [56]. Although comparison of epitopes from other proteins in the context of different human MHC class I alleles might allow further identification of bona fide dominant epitopes, the present study shows a useful approach to identify epitopes in complex pathogens like *T. cruzi* that are recognized by T cells restricted by different human class I alleles. This approach may be particularly useful in light of the recent demonstration of HLA promiscuity, i.e. that epitope presentation and CTL recognition in HIV-infected individuals may occur frequently in the context of alternative HLA class I alleles, with responses being detected in individuals not expressing the originally described restricting HLA allele [29].

Although, the possibility that IFN-*γ* responses were elicited by CD4^+^ T cells was not specifically addressed in the present study, the short 9 mer peptides used would unlikely be capable of binding class II MHC molecules. Of note, PBMC stimulated with ts epitopes did not induce IFN-g secretion by CD4^+^ T cells as determined by intracellular staining assays (data not shown). Moreover, in former studies, we confirmed that the IFN-g-producing T cells upon stimulation with HLA-A0201-binding peptides belonged to the CD8^+^ T cell compartment, as depletion of CD8^+^ T cells abolished the secretion of IFN-*γ* by PBMC [7] and staining with MHC class I tetramers detected CD8^+^ T cells specific for ts epitopes [25].

The present investigation was extended to determine the IFN-*γ* and IL-2 production profiles of T cells specific for ts epitopes from individuals at different clinical stages of chronic Chagas disease. A novel finding of our study is that subjects with chronic *T. cruzi* infection display a low prevalence of IL-2-secreting CD8^+^ and also CD4^+^ (as determined by stimulation with an amastigote lysate preparation) T cells specific for *T. cruzi*. Remarkably, the severe depletion of IL-2-secreting T cells was confined to those specific for *T. cruzi*, as higher frequencies of IL-2-secreting CD8^+^ T cells specific for influenza, a viral infection associated with antigen clearance, was found in these patients.

The presence of IL-2- and dual IL-2/IFN-γ-secreting CD4^+^ and CD8^+^ T cells has been associated with protracted antigen exposure and persistence with low antigen load and high proliferative capacity while IFN-γ-secreting T cells were associated with antigen persistence with high antigen load and poor proliferative capacity [41]–[43]. However, since in *T. cruzi* infection parasite load is extremely low in chronically infected individuals, the long-term parasite persistence rather than the high parasite load is likely to be responsible for driving the parasite specific T cell population to monofunctional IFN-γ-T cells with low self-renewal capacity.

In a mice model of chronic infection with the lymphocytic choriomeningitis virus, a hierarchical loss of different T-cell functions has been proposed with the production of IL-2 being the first function compromised followed by the ability to make tumor necrosis factor alpha, while IFN-γ production was most resistant to functional exhaustion [52]. Impaired IL-2 production is one of the main features of senescent T cells that lead to impaired proliferative capacity and decreased responsiveness to TCR stimulation as observed during aging [54],[57]. The loss of CD28 on CD8^+^ memory T cells found in chronic Chagas disease patients [8] is in agreement with the low levels of IL-2 secreting memory T cells since CD28 is essential for delivery of the second activation signal to T cells that leads to IL-2 gene transcription [58]. Moreover, CD4^+^ T cells producing IL-2 are also impaired in Chagas disease patients further compromising the T cell function in these patients.

This cytokine pattern of *T. cruzi*-specific CD8^+^ T cells along with the inverse correlation between IFN-γ responses and disease severity is in agreement with the phenotypic profile formerly found in this same cohort of patients in which the proportion of fully differentiated memory (CD45RA^−^CD27^−−^CD28^−−^) CD8^+^ T cells in the total memory CD8^+^ T cell population increases as disease becomes more severe [8]. We have also recently found that IFN-γ-secreting CD4^+^ T cells specific for *T. cruzi* are enriched in CCR7^−^CD122^−^CD127^−^ cells (Pérez et al., unpublished data) characteristic of long-term antigen stimulation.

As a whole, from these data we hypothesize that chronic stimulation with *T. cruzi* might lead to distinct stages of functional impairment and eventually to physical deletion of parasite-specific T cells, probably affecting the ability of the immune system to efficiently control the infection in these subjects with the possible consequence of accelerated disease progression.

We cannot rule out the possibility that the reduced T cell function observed in patients with advanced cardiomyopathy and heart failure may be a consequence of the heart failure itself rather than immunological attrition, although we are not aware of any mechanism that could account for such association. The monitoring of T cell responses during the natural course of *T. cruzi* infection would be required to further clarified this issue. We have mainly focused our study on *T. cruzi*-infected individuals without signs of heart disease in order to start building a map of T cell recognition and functionality in individuals capable of controlling the infection, thereby providing insight into what constitutes an effective immune response in *T. cruzi* infection,

The set of class I MHC restricted peptides identified herein will be useful for the measurement of the frequency and functional activity of *T. cruzi*-specific CD8^+^ T cells in *T. cruzi*-infected individuals and should facilitate the monitoring of immune competence and changes in infection and disease status in individuals with chronic Chagas disease.

## Supporting Information

Alternative Language Abstract S1 Translation of the Abstract into Spanish by Susana Laucella
(0.02 MB DOC)
