# Supplementary material for: HLA Class I-T Cell Epitopes from trans-Sialidase Proteins Reveal Functionally Distinct Subsets of CD8+ T Cells in Chronic Chagas Disease
Source: PLoS Negl Trop Dis. 2008 Sep 3;2(9):e288. doi: 10.1371/journal.pntd.0000288 (PMC2565697; doi:10.1371/journal.pntd.0000288)
Supplement: Alternative Language Abstract S1 — Translation of the Abstract into Spanish by Susana Laucella (0.02 MB DOC) [file pntd.0000288.s001.doc]

Translation of the Abstract into Spanish by Susana Laucella.

**Epitopes derivados de transialidasas revelan subpoblaciones de linfocitos T CD8^+^ con diferente capacidad funcional en la enfermedad de Chagas crónica.**

**Antecedentes.** En trabajos previos, identificamos un grupo de epitopes derivados de la familia de las transialidasas del *Trypanosoma cruzi* con capacidad de unión a la molécula de histocompatibilidad HLA-A020.1 como blanco de la respuesta celular T CD8^+^. **Materiales y Resultados.** En el presente estudio**,** presentamos la identificación de epitopes codificados por la misma familia de genes de las transialidasas con capacidad para unirse a distintos alelos representativos de los supertipos clase I más comunes en la población. En base a algoritmos informáticos, se identificaron un total de 1001 epitopes que potencialmente podrían unirse a los supertipos HLA A01, A02, A03, A24, B7 y B44. De estos, se seleccionaron 96 péptidos codificados por múltiples genes para evaluar su capacidad para estimular una respuesta T CD8^+^ utilizando células mononucleares periféricas de pacientes crónicamente infectados por *T. cruzi* independientemente del haplotipo de los mismos. Los péptidos con capacidad de unión al HLA-A02 fueron los más frecuentemente reconocidos en la población infectada seguido por péptidos con alta afinidad de unión a la molécula HLA-A03 y A24. Las respuesta T CD8^+^ hacia estos epitopes “promiscuos” reveló que el compartimento T CD8^+^ específico para *T. cruzi* presenta como perfil prevalente células capaces de secretar sólo IFN-g y una muy baja frecuencia de linfocitos productores de IL-2 o linfocitos con función dual productores de IL-2 e IFN-γ. **Conclusiones.** Este estudio identificó un grupo de epitopes con potencial utilidad para el monitoreo de inmunocompetencia y de cambios asociados a la progresión de la enfermedad en individuos crónicamente infectados con *T. cruzi.*
